# Supplementary material for: Segregation, integration, and balance of large-scale resting brain networks configure different cognitive abilities
Source: Proc Natl Acad Sci U S A. 2021 May 31;118(23):e2022288118. doi: 10.1073/pnas.2022288118 (PMC8201916; doi:10.1073/pnas.2022288118)
Supplement: Supplementary File [file pnas.2022288118.sapp.pdf]

Supplementary Information for

Segregation, integration and balance of large-scale resting brain networks configure different cognitive abilities

Rong Wang, Mianxin Liu, Xinhong Cheng, Ying Wu, Andrea Hildebrandt, Changsong Zhou

Andrea Hildebrandt, Changsong Zhou.
E-mail: andrea.hildebrandt@uni-oldenburg.de (A.H.); cszhou@hkbu.edu.hk (C.Z.)

This PDF file includes:

Supplementary text
Figs. S1 to S12 (not allowed for Brief Reports)
Tables S1 to S2 (not allowed for Brief Reports)
Legend for Dataset S1
SI References

Other supplementary materials for this manuscript include the following:

Dataset S1

Supporting Information Text

Supplementary Methods

Data processing. We used the MRI data preprocessed by the HCP through the well-established and -accepted Minimal Processing Pipeline (MPP) (1). The MPP performed several conventional processing steps, including spatial artifact/distortion removal, surface generation, cross-modal registration, and alignment to standard space. The output MRI data were thus artifact removed and spatial variation registered across individuals and modalities. More details on these processing steps are described and discussed in Ref. (1). The preprocessed resting-state fMRI data were converted to surface space ("CIFTI" format), which consists of 91282 cortical and subcortical grayordinates with a resolution of 2 mm (2). Since the global component of the fMRI fluctuations measured during the resting state is tightly coupled with the underlying neural activity, the use of global signal regression as a pre-processing step in resting-state fMRI analyses remains controversial and is not universally recommended (3). Therefore, the global whole-brain signal was not removed in this analysis, and some fMRI signals may inevitably originate from sources other than brain. Furthermore, we parcellated the brain into 360 regions according to the multimodal parcellation (MMP) atlas (4) so that blood-oxygen level dependent (BOLD) time series for each region could be obtained by averaging voxel signals located within regions. To remove slow drifts induced by the scanner hardware and possibly high frequency noise, the well-accepted filtering band-pass of 0.01-0.1Hz (5–8) was applied in fMRI data to extract the "low frequency fluctuations" that was believed to be most relevant to neural activity in BOLD signal in previous literatures (9). Preprocessing was completed separately for each session.

Probabilistic tractography was performed on the DTI data to trace the white matter fibers bridging brain regions with the widely accepted default settings in the FSL software (version 5.0.9, http://www.fmrib.ox.ac.uk/fsl/) (4, 10, 11). Thus, structural connectivity among 360 regions was extracted for each individual. First, the 360 MMP regions defined in the standard space was projected into individual cortical surfaces in the diffusion space by using the Connectome Workbench (2). Probabilistic tractography was then performed among pairs of brain regions with the FSL software (12, 13). During pair-wise fiber tracing, one region was defined as the seed area to start the tracing and the other as the target, and vice versa. Each voxel of the seed region will send out a total of 5000 streamlines. Streamline propagation were performed with a step length of 0.5 mm and a maximum step of 2000. The propagation will terminate when the brain pial surface was encountered or the curvature of the next step is too high compared with the previous step (threshold was set to default value 0.2 in FSL). In addition, individual fractional anisotropy (FA) maps generated using FSL based on the pre-processed DTI data were also used to set termination threshold for the fiber tracing. Fiber streamline propagation would cease when reaching a voxel with an FA value less than 0.1. After fiber tracing, the directional connective probability p_{ij} from a seed region i to a target region j can be calculated as the ratio between the number of streamlines that reached the target region and the total number of streamlines initiated from the seed region (5000 multiplies the number of voxels in the seed region) (12). Based on the calculated directional connective probability, we further defined the structural connectivity weight w_{ij}, by calculating the reciprocal averages of the connective probabilities (i.e. $w_{ij} = (p_{ij} + p_{ji})/2$). A higher w_{ij} indicated stronger structural connectivity between regions i and j.

Gaussian linear diffusion model. The emergent macroscopic firing activities of neuronal population in specific brain regions may not strongly depend on the detailed knowledge of interactions between individual neurons and instead obey laws relating to their ensemble dynamics. In local neural populations in the brain when receiving no structured external inputs, the spontaneous activities are highly variable due to a balance of excitation and inhibition (14). In the excitation-inhibition balanced regime, neural firing is driven by fluctuations and is highly stochastic (14). Without considering detailed nonlinear neural properties (15), we can approximate the variable dynamics as noise-driven fluctuation and relaxation around a baseline state (15–17), which is described as:

$$\dot{x}_i = -ax_i + \sqrt{2a\sigma^2}\xi_i \qquad [1]$$

where x_i represents the neural activities of the ith neural population, a controls the relaxation rate of neural activities, σ is the noise strength and ξ_i is the Gaussian white noise with zero mean and unit variance. The values of σ and a have no effect on the functional connectivity (FC) matrix over long enough time ($T \gg 1/a$) and we set $a = 1$, σ=1 without loss of generality. Consequently, the collective dynamics of coupled neural populations can be approximately modeled as:

$$\dot{x}_i = -x_i + \sum_{j=1}^{N} f(c, A_{ij}, x_j, x_i) + \sqrt{2}\xi_i \qquad [2]$$

Here, f is the coupling function, c is the coupling strength between neural populations and A is the brain SC matrix. The coupling function reflects the impact of the incoming neural activity on the local region. Since the local region is composed of excitation and inhibition which are balanced, the input thus does not simply enhance the overall activity of the local region, and the effect would naturally depend on the activation level of the local region, e.g. through diffusion (15). We thus consider the simplest linear diffusion coupling between neural populations and rewrite Eq. 2 as:

$$\dot{x}_i = -x_i + c\sum_{j=1}^{N} A_{ij}(x_j - x_i) + \sqrt{2}\xi_i \qquad [3]$$

The covariance of this Gaussian model can be analytically estimated by averaging over the states produced by an ensemble of noise (18, 19), and the Eq. 3 can be linearized as:

$$x_i = c\sum_{j=1}^{N} A_{ij}(x_j - x_i) + \sqrt{2}\xi_i \tag{4}$$

with the matrix form

$$X = -cHX + \sqrt{2}\xi \tag{5}$$

Here H is the Laplace matrix. Solving the Eq. 5, we have

$$X = \frac{1}{1+cH}\sqrt{2}\xi = \sqrt{2}Q\xi \tag{6}$$

and the covariance matrix is consequently calculated as:

$$COV = \left\langle XX^T \right\rangle = 2Q\left\langle \xi\xi^T \right\rangle Q^T = 2QQ^T \tag{7}$$

as well as the simulated FC matrix C:

$$C_{ij} = \frac{COV_{ij}}{\sqrt{COV_{ii}COV_{jj}}} \tag{8}$$

Thus, we obtain the simulated stable FC matrices for long enough time.

Cohen's d effect size. Cohen's d gives the effect size for the comparison between two means:

$$d = \frac{m_1 - m_2}{\sqrt{\frac{(n_1-1)sd_1^2+(n_2-1)sd_2^2}{n_1+n_2-2}}} \tag{9}$$

Here, m_1 and m_2 are two means in two groups, n_1 and n_2 are group sizes, sd_1 and sd_2 are the corresponding standard deviation. This Cohen's d may have negative and positive values due to the setting of groups as m_1 or m_2, and here the absolute Cohen's d was used. Cohen suggested that $d < 0.2$ reflects a trivial mean difference; $d \geq 0.2$ reflects a small effect size, $d \geq 0.5$ represents a medium effect size and $d \geq 0.8$ indicates a large effect size.

Supplementary Figures

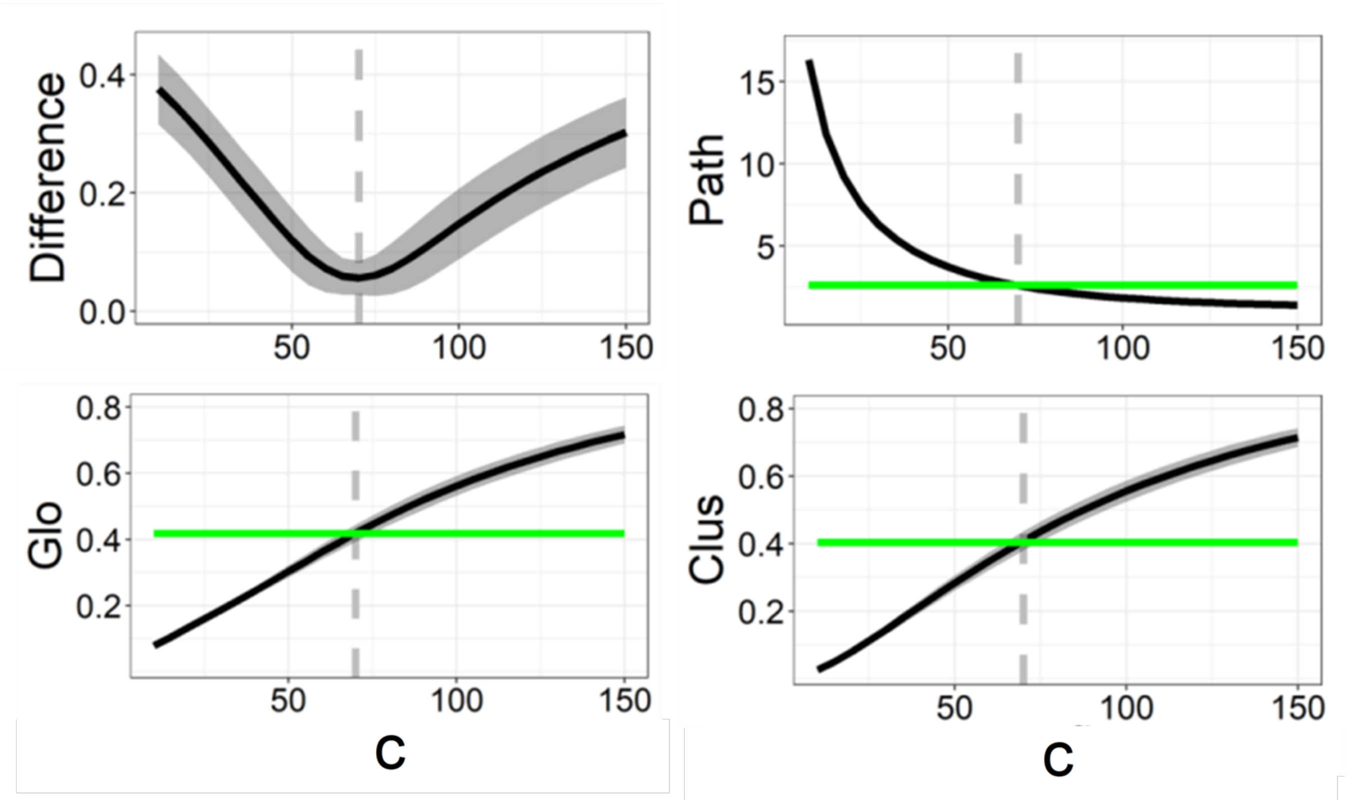

Fig. S1. Comparisons of topological properties between real and simulated FC networks. At the critical coupling $c = 70$, the simulated and real stable FC networks have the minimal difference of regional degrees, equal characteristic path length, global efficiency and clustering coefficient. These graph-based measures were calculated using the BCT toolbox (see Ref. (20) for detailed equations).

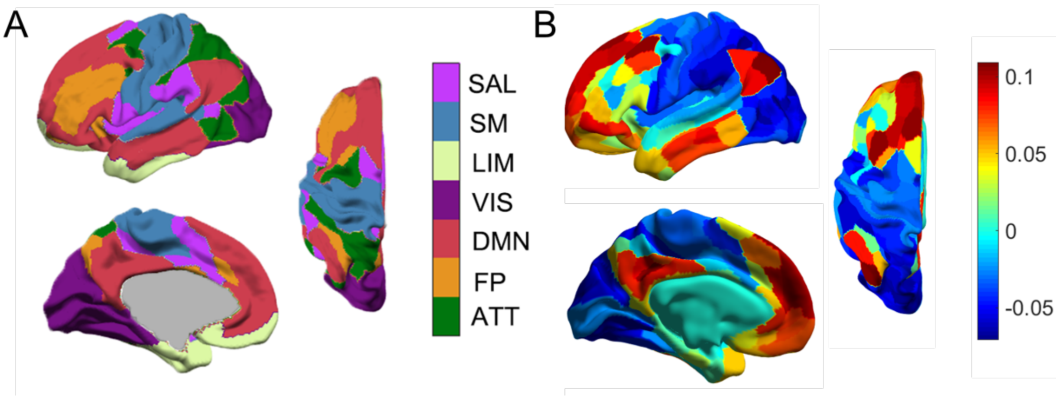

Fig. S2. Brain mappings of functional subsystems and the second functional mode. (A) Seven functional subsystems (21). SAL: salience network; SM: somatomotor network; LIM: limbic network; VIS: visual network; DMN: default mode network; FP: frontoparietal network; ATT: attention network. (B) Brain mapping of the second functional mode. Since the intrinsic differences between our NSP method and classical methods, our hierarchical module partition is, in principle, not expected to be completely matched with the known functional networks (e.g., Yeo et al functional subsystems (21)). But the eigenmodes are the basic patterns of brain activations and all the previous detected functional subsystems by other methods can be reproduced by the combination of several significant modes (22)). For example, in the 2nd level of brain FC network, the coactivated regions within a module form a functional pattern very similar to the DMN. Thus, the DMN is significantly represented by this level, but also involves other levels.

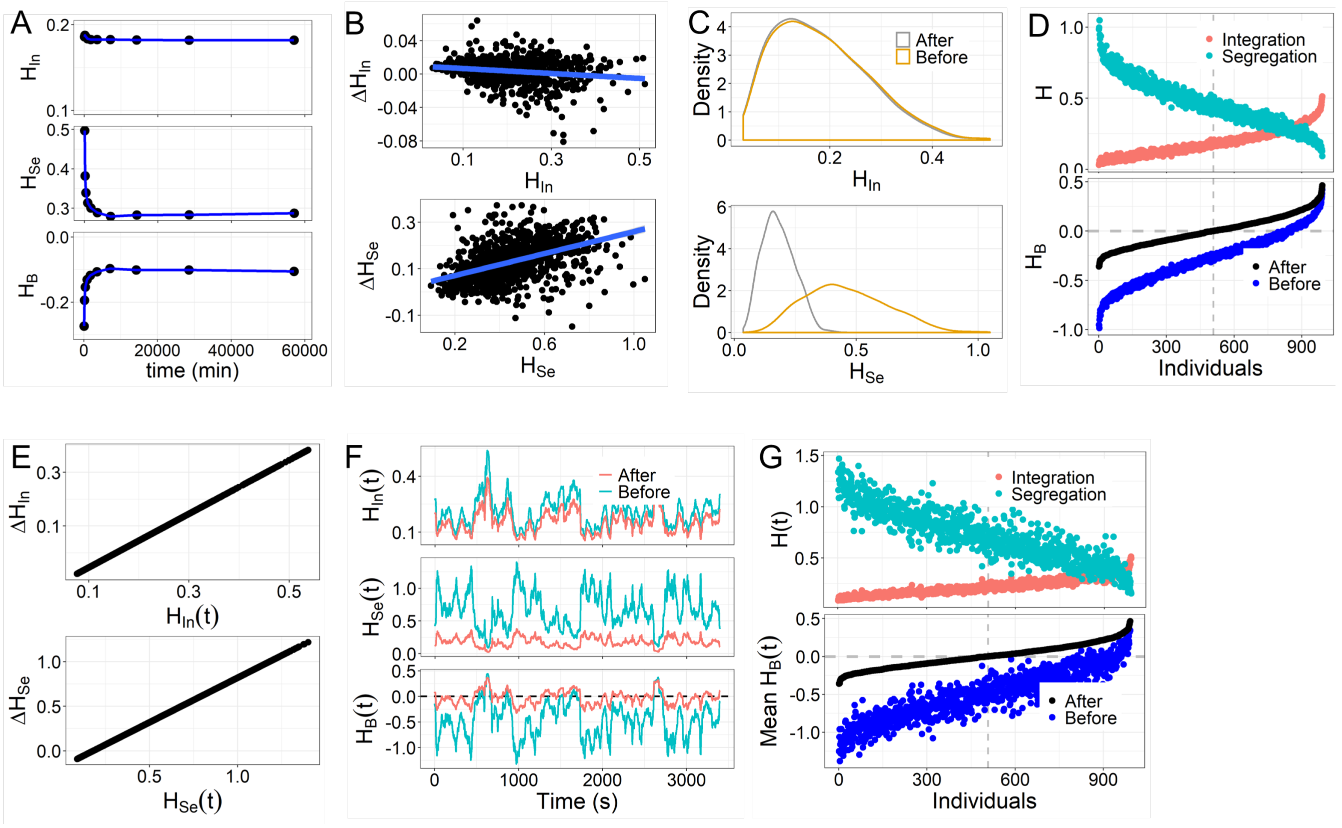

Fig. S3. Calibration process of segregation and integration components. (A) The integration component H_{In}, segregation component H_{Se} and balance indicator H_B for different lengths of fMRI time series, achieved by concatenating time series of different numbers of individuals. The length of fMRI time series mainly affects the segregation component H_{Se} and has little effect on the integration component H_{In}. This phenomenon induces deviation of the group-averaged brain from a balanced state apparently toward segregated state. Thus, the segregation and integration components in individuals need to be calibrated (group calibration). (B) To determine the calibration rule, we compared the H_{In} (upper panel) and H_{Se} (lower panel) difference between FC networks computed from the single session and the concatenated four sessions for each individual, wherein H_{In} and H_{Se} in x-axis are from the static FC networks with concatenated four sessions. It is apparent that the length of fMRI series mainly affects the segregation component, and ΔH_{Se} is approximately proportional to H_{Se}, indicating that H_{Se} in the segregated brain is more greatly affected by different lengths. Thereby, we adopted the proportional calibration for H_{In} and H_{Se}, which is an approximate method to obtain the fMRI length-independent individual segregation and integration components. (C) Individual static FC networks from four concatenated sessions. The original H_{In} and H_{Se} (marked by the Before) in the group are calibrated to the balanced state (marked by the After) with the proportional calibration. This is because we have identified the theoretical and empirical evidence of brain balance from long enough time series. It is apparent that the calibration mainly changes H_{Se} and has little effect on H_{In}. (D) Upper panel: original H_{In} and H_{Se}; lower panel: original and calibrated H_B. After the calibration, individual rankings of H_B are fixed and independent on time series length, which is the baseline for the calibration in dynamic analysis. (E) Because the temporal FC network is computed from a short sliding window (59.76s) and more negative connectivity is excluded, the individual rankings change. To maintain the individual ranking, the mean values of $H_{In}(t)$ and $H_{Se}(t)$ for each individual are respectively calibrated to its static H_{In} and H_{Se} (individual calibration). To determine the calibration rule within individuals, we compared the difference between H_{In} and $H_{In}(t)$ (i.e., $\Delta H_{In}(t) = H_{In}(t) - H_{In}$, upper panel, see lower panel for the difference between H_{Se} and $H_{Se}(t)$, i.e., $\Delta H_{Se}(t) = H_{Se}(t) - H_{Se}$) for each individual, wherein the H_{In} and H_{Se} are the calibrated static results. Since the linear relationship between $\Delta H_{In}(t)$ and $H_{In}(t)$ (or between $\Delta H_{Se}(t)$ and $H_{Se}(t)$) always exist for individuals, the proportional calibration (i.e., $H_{Se}^{'}(t) = H_{Se}(t)\frac{H_{Se}}{\langle H_{Se}(t)\rangle}$ and $H_{In}^{'}(t) = H_{In}(t)\frac{H_{In}}{\langle H_{In}(t)\rangle}$) is also adopted in the individual calibration process, where the $\langle\rangle$ represents the average across time. (F) Comparison between original (before) and calibrated (after) $H_{In}(t)$, $H_{Se}(t)$ and $H_B(t)$ in one individual. The individual calibration pushes $H_B(t)$ to move up and thus the length-independent dynamic properties are obtained. (G) For the dynamic FC networks, the mean values of $H_{In}(t)$, $H_{Se}(t)$ for each individual are respectively calibrated to the corresponding static H_{In} and H_{Se} with the individual calibration. Thus, the individual rankings in dynamic analysis are kept as that in static analysis, and the static and dynamic results are independent on the fMRI length.

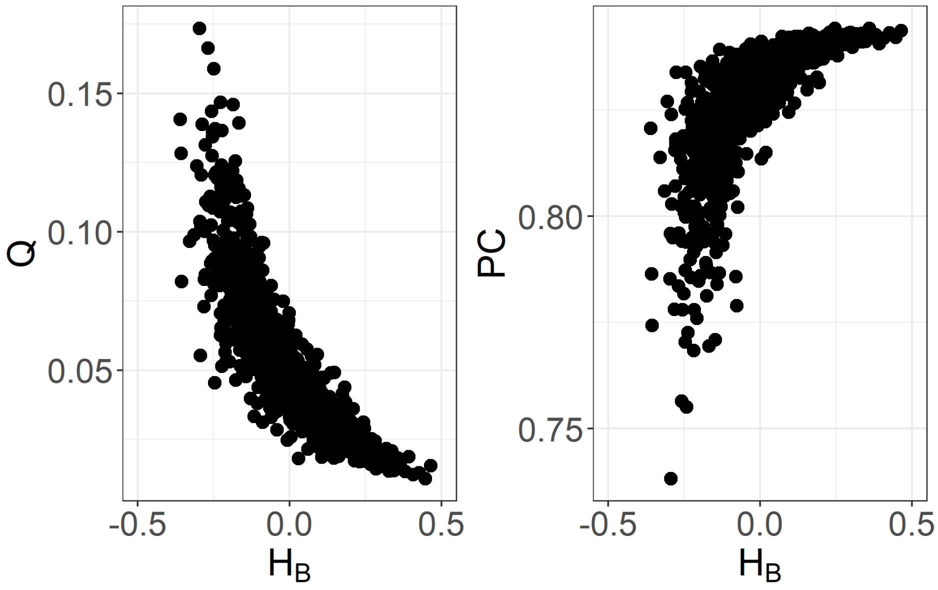

Fig. S4. Comparisons between H_B and graph-based network measures. The modularity Q and participation coefficient PC were calculated from the partition of seven functional subsystems. We observed a strong correlation between H_B and modularity, but no explicit association with the participation coefficient. This result further proves that on the one hand our measures can reflect the segregation and integration, and on the other hand our measures are intrinsically different as compared with graph-based network measures.

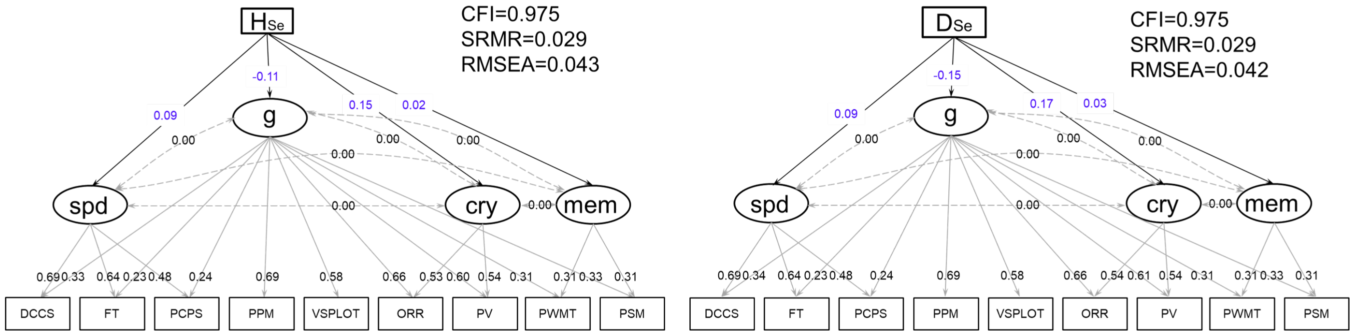

Fig. S5. Schematic representation of SEM exploring linear relationships between cognitive abilities and brain measures. DCCS–Dimensional Change Card Sort, FT–Flanker Task, PCPS–Pattern Completion Processing Speed, PPM–Penn Progressive Matrices, VSPLOT–Variable Short Penn Line Orientation Test, ORR–Oral Reading Recognition, PV–Picture Vocabulary, PWMT–Penn Word Memory Test, PSM–Picture Sequence Memory.

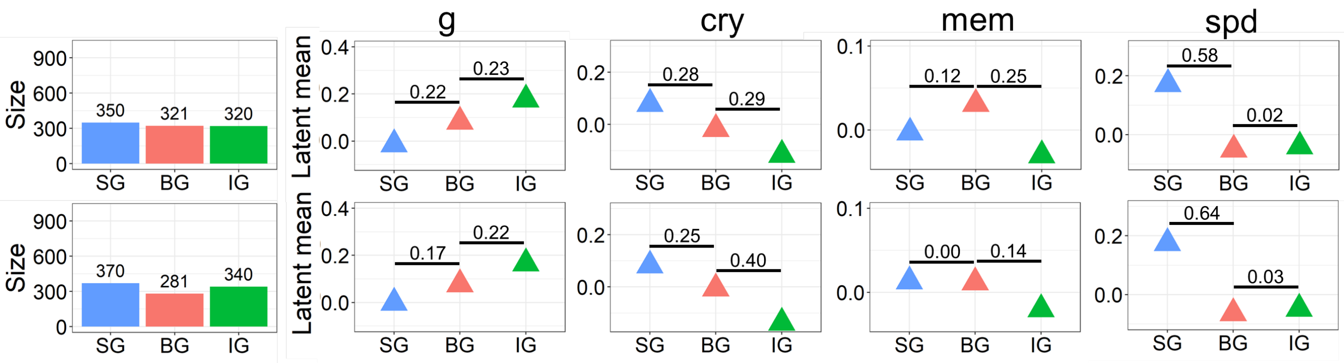

Fig. S6. Multiple group SEMs according to three subgroups with different group sizes. These results provide further evidence that better crystallized intelligence and processing speed rely on higher segregation, better general cognitive ability is related to higher integration, and better memory is supported by a balance between segregation and integration.

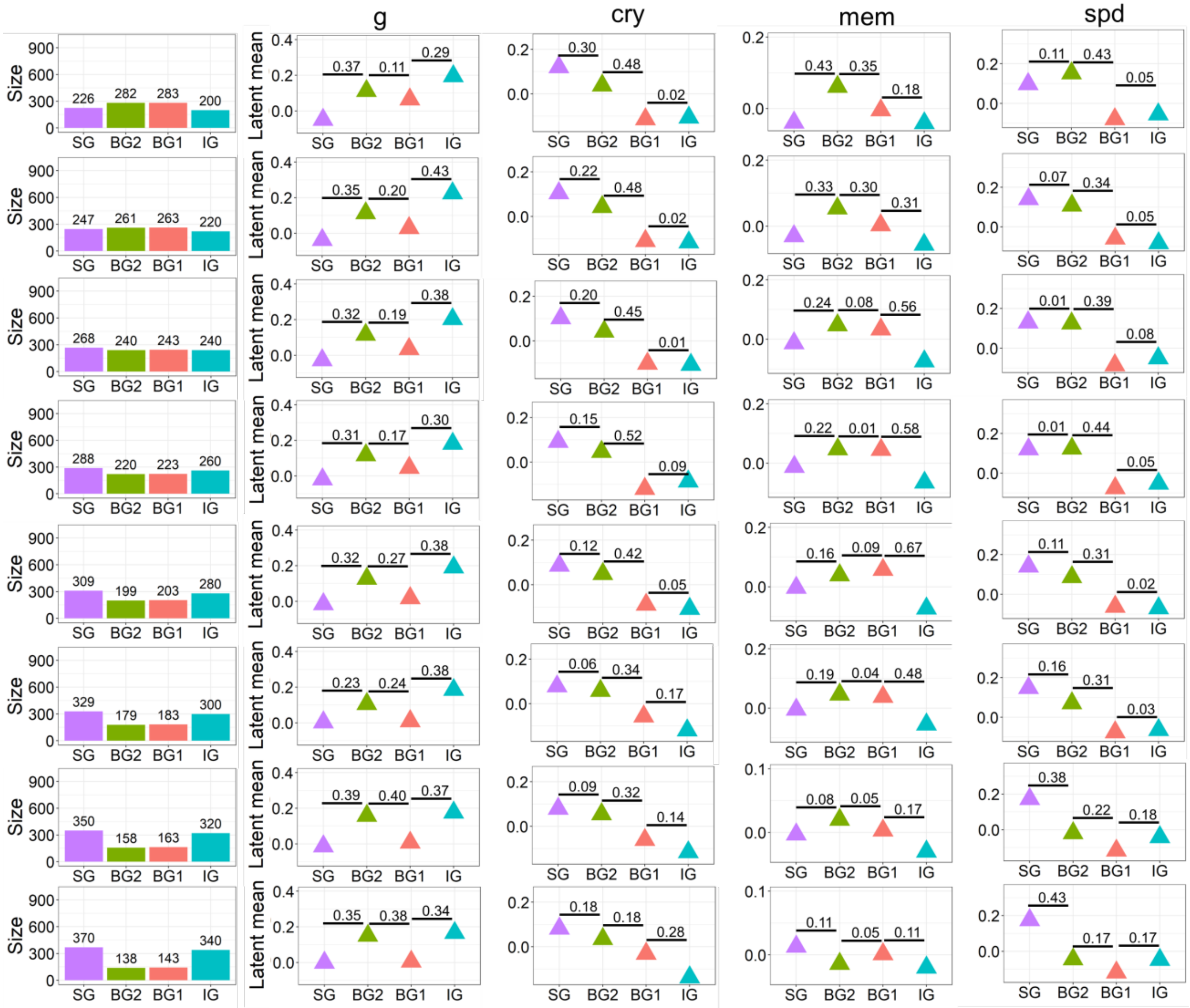

Fig. S7. Results of multiple group SEMs in four subgroups. Here, the BG group is further divided into two subgroups with BG1 (i.e., $H_B > 0$) and BG2 (i.e., $H_B < 0$). These results provide further support for the robustness of our main claims.

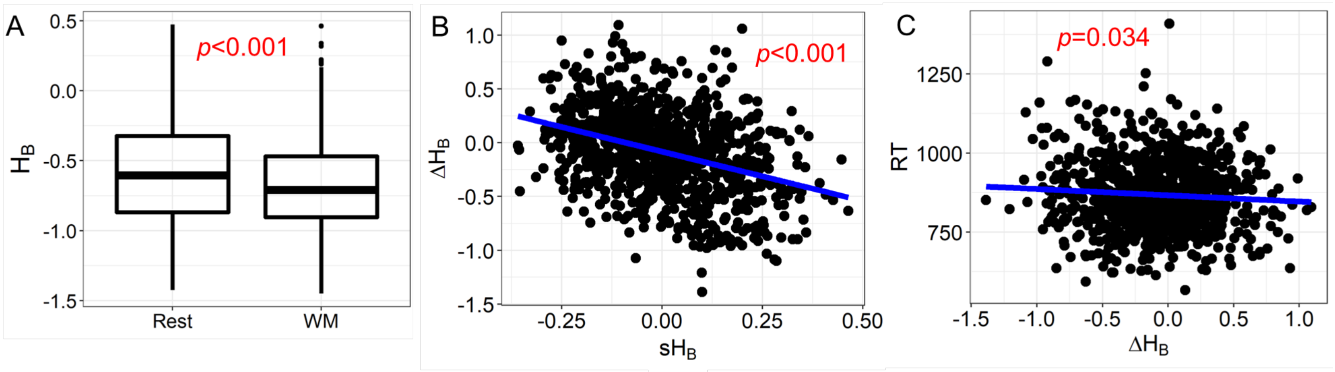

Fig. S8. Segregation and integration in task fMRI and association to performance outcome. In the working memory (WM) task, the 0-back and 2-back blocks randomly appeared and the total task fMRI length was 608 points (about 437.76s) across two scans. As a comparison, the resting-state fMRI also contained 608 points for each individual. Then, the static FC was computed with the Pearson correlation coefficient for the rest and WM groups. (A) The boxplots of H_B values at resting and task states where the H_B was not calibrated. The significantly decreased H_B for the WM group (two-sample t-test, $p < 0.001$) indicates that WM task need higher segregation. (B) The individual change of H_B induced by WM , comparing to the resting state. Here, the sH_B is the fMRI length-independent measure (i.e., H_B in our paper) that reflects the real state of individual brains at rest. The negative correlation indicates that for the resting brain with higher segregation (i.e., negative sH_B), it has less change during WM task. Thus, the WM task needs higher segregation, and the brain with higher segregation at rest is more efficient in switching to the task state with less change of H_B. (C) The relationship between the task reaction time (RT) and the change of H_B. Less change of H_B corresponds to smaller RTs, indicating that the brain's more efficient switching from resting to task states generates better task performance, matching with previous studies (23–27). Therefore, brains with higher segregation at rest exhibit a higher efficiency in switching to WM state that needs higher segregation, and higher switching efficiency corresponds to better task performance. This result provides a preliminary support to the our hypothesis: resting brains tending toward segregation are more efficient to switch to task states that need higher segregation. However, task-fMRI data systematically covering a broad range of tasks are needed to test the hypothesis comprehensively. Even the HCP contains 7 tasks during which fMRI was measured, these tasks cover a broad range of processes from the emotion domain as well. Because the cognitive domains reflected in the SEM model are not covered with task-fMRI data in the HCP, there would be no potential symmetry between the psychometric and task fMRI measurements if we extended our aim from analyzing resting-state fMRI also to task-fMRI data.

A

	S1	S2	S3	S4
S1		0.59	0.61	0.54
S2			0.57	0.60
S3				0.58
S4				

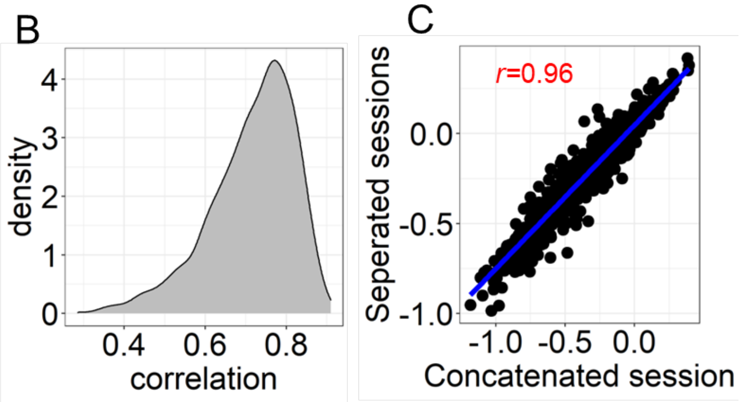

Fig. S9. Reliabilities of FC and H_B. (A) Correlations of FC among four sessions of each individual, averaged across all individuals. The individual static FC was first calculated for each session, then for each individual the correlation of FC was calculated between any two sessions. Finally, the correlation between any two sessions was averaged across all individuals. The overall correlation across all sessions and individuals is 0.58. S1 – first session, S2 – second session, S3 – third session and S4 – fourth session. (B) The distribution of FC correlations between two days. The fMRI time series were first respectively concatenated within each day, and then individual static FC was calculated. The correlation between FCs from two days was obtained for each individual and the distribution of the correlations is visualized as a density plot. The average correlation across all individuals is 0.72. (C) The correlation of H_B based on four concatenated sessions and the average H_B from four separated sessions is visualized. Note that the H_B for four separated sessions was first separately calculated for the sessions and then averaged within each individual.

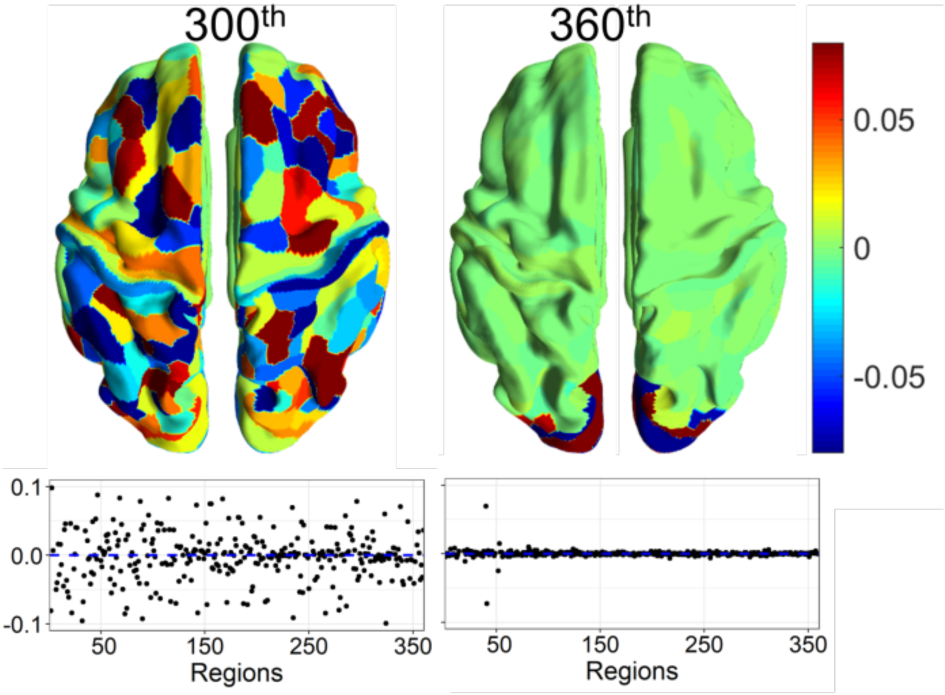

Fig. S10. Examples of higher-order functional modes. In higher-order modes, regions are independently activated, producing completely segregated process.

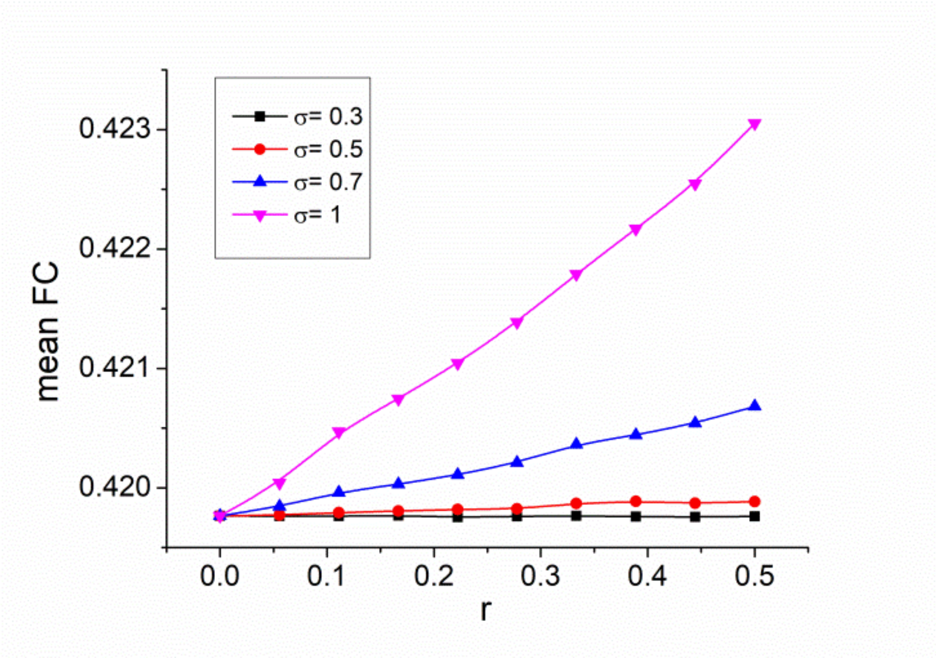

Fig. S11. Robustness in the estimate of the modeled FC with respect to noise in weak links. In the Gaussian model with the critical coupling strength $c = 70$, we modified the weakest links (with a ratio r among all the detected links), by adding noise to these links as $p_{ij} = p_{ij}(1 + \sigma\xi)$, where ξ is Gaussian noise with zero mean and one standard deviation 1.0, and σ is the relative strength of noise. After this modification, those negative SC values were set to zero and the SC matrix was symmetrized. Larger r or σ can increase the mean FC, but the change is very small (in the 3rd digit). For example, while $50\%(r = 0.5)$ weakest SC was changed by a relatively strong noise level up to $\sigma = 0.5$, the mean FCs are nearly the same. Therefore, the simulated FC is robust to the DTI data processing.

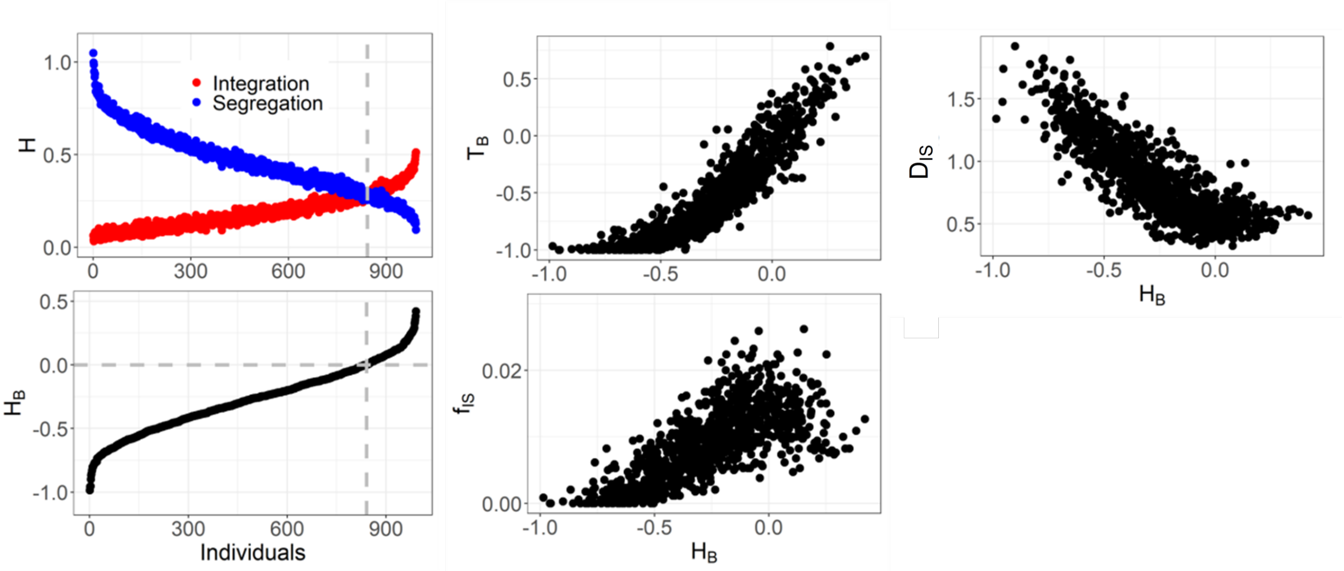

Fig. S12. Main results for uncalibrated results. Individuals with $H_B \approx 0$ under the uncalibrated results, still have the $T_B \approx 0$, maximal f_{IS} and minimal D_{IS}, but the phenomenon is not so pronounced as the calibrated results (Fig. 4). Therefore, it can be concluded that the calibration process does not affect the dynamical results, but can greatly highlight these properties.

Table S1. Linear relationships between brain measures and cognitive abilities. Three kinds of brain measures are shown: calibrated and uncalibrated measures based on hierarchical modules in FC networks, graph-based network measures including modularity and participation coefficients at a single level. Here, the modularity and participation coefficient are calculated based on seven functional subsystems. The degree (D), characteristic path length (PL) and global clustering coefficient (CC) are also calculated to facilitate comparisons with previous studies. It becomes obvious that our measures based on hierarchical modules are more effective in predicting cognitive abilities. The significant results are highlighted by grey color.

Calibrated measures					
β (p-value)	g	cry	mem	spd	Model fit indices
H_B	0.087(0.037)	-0.125(0.016)	-0.029(0.715)	-0.097(0.016)	CFI=0.975, RMSEA=0.042, SRMR=0.028
H_{Se}	-0.113(0.007)	0.148(0.005)	0.016(0.834)	0.093(0.022)	CFI=0.975, RMSEA=0.043, SRMR=0.029
D_{Se}	-0.155(0.000)	0.166(0.002)	0.032(0.692)	0.089(0.028)	CFI=0.975, RMSEA=0.042, SRMR=0.029
H_{In}	0.064(0.124)	-0.103(0.045)	-0.036(0.642)	-0.097(0.017)	CFI=0.975, RMSEA=0.042, SRMR=0.028
T_{In}	0.063(0.127)	-0.116(0.025)	-0.055(0.484)	-0.09(0.027)	CFI=0.974, RMSEA=0.043, SRMR=0.029
f_{IS}	0.034(0.413)	-0.086(0.093)	-0.106(0.184)	-0.071(0.097)	CFI=0.976, RMSEA=0.042, SRMR=0.028
D_{In}	0.048(0.252)	-0.088(0.085)	-0.003(0.964)	-0.089(0.028)	CFI=0.975, RMSEA=0.042, SRMR=0.029
D_{IS}	-0.035(0.398)	0.050(0.330)	0.055(0.483)	-0.028(0.486)	CFI=0.974, RMSEA=0.043, SRMR=0.029
Uncalibrated measures					
H_B	0.097(0.019)	-0.135(0.010)	-0.024(0.759)	-0.097(0.017)	CFI=0.975, RMSEA=0.042, SRMR=0.029
H_{Se}	-0.113(0.007)	0.148(0.005)	0.016(0.834)	0.093(0.022)	CFI=0.975, RMSEA=0.043, SRMR=0.029
D_{Se}	-0.095(0.022)	0.124(0.017)	0.100(0.211)	0.074(0.070)	CFI=0.974, RMSEA=0.043, SRMR=0.029
Graph-based network measures					
Q	-0.039(0.352)	0.113(0.028)	0.088(0.266)	0.092(0.022)	CFI=0.974, RMSEA=0.043, SRMR=0.029
PC	0.038(0.356)	-0.097(0.058)	-0.083(0.295)	-0.084(0.037)	CFI=0.973, RMSEA=0.044, SRMR=0.029
D	0.066(0.110)	-0.116(0.024)	-0.052(0.512)	-0.098(0.015)	CFI=0.974, RMSEA=0.043, SRMR=0.028
CC	0.067(0.105)	-0.117(0.023)	-0.057(0.47)	-0.098(0.015)	CFI=0.974, RMSEA=0.043, SRMR=0.028
PL	-0.077(0.065)	0.135(0.009)	0.06(0.445)	0.094(0.020)	CFI=0.974, RMSEA=0.043, SRMR=0.028

Table S2. Detailed information of nine specific cognitive performance tasks.

Full Display Name	Assessment	Column Header
NIH Toolbox Picture Sequence Memory Test	Episodic Memory (Picture Sequence Memory)	PSM
NIH Toolbox Dimensional Change Card Sort Test	Executive Function/Cognitive Flexibility (Dimensional Change Card Sort)	DCCS
NIH Toolbox Flanker Inhibitory Control and Attention Test	Executive Function/Inhibition (Flanker Task)	FT
Penn Progressive Matrices: Number of Correct Responses	Fluid Intelligence (Penn Progressive Matrices)	PPM
NIH Toolbox Oral Reading Recognition Test	Language/Reading Decoding (Oral Reading Recognition)	ORR
NIH Toolbox Picture Vocabulary Test	Language/Vocabulary Comprehension (Picture Vocabulary)	PV
NIH Toolbox Pattern Comparison Processing Speed Test	Processing Speed (Pattern Completion Processing Speed)	PCPS
Variable Short Penn Line Orientation: Total Number Correct	Spatial Orientation (Variable Short Penn Line Orientation Test)	VSPLOT
Penn Word Memory Test: Total Number of Correct Responses	Verbal Episodic Memory (Penn Word Memory Test)	PWMT

SI Dataset S1 (psychometrics_cov.csv)

The covariance matrix, as well as the mean and standard deviation vectors of all task performance indicators.

References

1. MF Glasser, et al., The minimal preprocessing pipelines for the human connectome project. *Neuroimage* **80**, 105–124 (2013).
2. DC Van Essen, et al., The wu-minn human connectome project: an overview. *Neuroimage* **80**, 62–79 (2013).
3. ML Schölvinck, A Maier, QY Frank, JH Duyn, DA Leopold, Neural basis of global resting-state fmri activity. *Proc. Natl. Acad. Sci.* **107**, 10238–10243 (2010).
4. MF Glasser, et al., A multi-modal parcellation of human cerebral cortex. *Nature* **536**, 171 (2016).
5. JM Shine, MJ Aburn, M Breakspear, RA Poldrack, The modulation of neural gain facilitates a transition between functional segregation and integration in the brain. *Elife* **7**, e31130 (2018).
6. F Sobczak, Y He, TJ Sejnowski, X Yu, Predicting the fmri signal fluctuation with recurrent neural networks trained on vascular network dynamics. *Cereb. Cortex* **31**, 826–844 (2021).
7. JR Whittaker, ID Driver, M Venzi, MG Bright, K Murphy, Cerebral autoregulation evidenced by synchronized low frequency oscillations in blood pressure and resting-state fmri. *Front. Neurosci.* **13**, 433 (2019).
8. R Wang, et al., Hierarchical connectome modes and critical state jointly maximize human brain functional diversity. *Phys. Rev. Lett.* **123**, 038301 (2019).
9. B Biswal, F Zerrin Yetkin, VM Haughton, JS Hyde, Functional connectivity in the motor cortex of resting human brain using echo-planar mri. *Magn. Reson. Med.* **34**, 537–541 (1995).
10. X Liu, A Hildebrandt, K Meyer, W Sommer, C Zhou, Patterns of individual differences in fiber tract integrity of the face processing brain network support neurofunctional models. *Neuroimage* **204**, 116229 (2020).
11. M Liu, X Liu, A Hildebrandt, C Zhou, Individual cortical entropy profile: Test-retest reliability, predictive power for cognitive ability, and neuroanatomical foundation. *Cereb. Cortex Commun.* (2020).
12. TE Behrens, HJ Berg, S Jbabdi, MF Rushworth, MW Woolrich, Probabilistic diffusion tractography with multiple fibre orientations: What can we gain? *Neuroimage* **34**, 144–155 (2007).
13. TE Behrens, et al., Characterization and propagation of uncertainty in diffusion-weighted mr imaging. *Magn. Reson. Med.* **50**, 1077–1088 (2003).
14. C Van Vreeswijk, H Sompolinsky, Chaos in neuronal networks with balanced excitatory and inhibitory activity. *Science* **274**, 1724–1726 (1996).
15. F Abdelnour, HU Voss, A Raj, Network diffusion accurately models the relationship between structural and functional brain connectivity networks. *Neuroimage* **90**, 335–347 (2014).
16. G Zamora-Lopez, Y Chen, G Deco, ML Kringelbach, C Zhou, Functional complexity emerging from anatomical constraints in the brain: the significance of network modularity and rich-clubs. *Sci. Rep.* **6**, 38424 (2016).
17. N Brunel, XJ Wang, Effects of neuromodulation in a cortical network model of object working memory dominated by recurrent inhibition. *J. Comput. Neurosci.* **11**, 63–85 (2001).
18. A Papoulis, SU Pillai, *Probability, random variables, and stochastic processes.* (Tata McGraw-Hill Education, New York), Fourth edition, (2002).
19. G Tononi, O Sporns, GM Edelman, A measure for brain complexity: relating functional segregation and integration in the nervous system. *Proc. Natl. Acad. Sci.* **91**, 5033–5037 (1994).
20. M Rubinov, O Sporns, Complex network measures of brain connectivity: Uses and interpretations. *Neuroimage* **52**, 1059–1069 (2010).
21. BT Yeo, et al., The organization of the human cerebral cortex estimated by intrinsic functional connectivity. *J. Neurophysiol.* **106**, 1125–1165 (2011).
22. S Atasoy, I Donnelly, J Pearson, Human brain networks function in connectome-specific harmonic waves. *Nat. Commun.* **7**, 10340 (2016).
23. K Machida, KA Johnson, Integration and segregation of the brain relate to stability of performance in children and adolescents with varied levels of inattention and impulsivity. *Brain Connect.* **9**, 711–729 (2019).
24. F Schmitz, O Wilhelm, Modeling mental speed: Decomposing response time distributions in elementary cognitive tasks and correlations with working memory capacity and fluid intelligence. *J. Intell.* **4**, 13 (2016).
25. M Girn, C Mills, K Christoff, Linking brain network reconfiguration and intelligence: Are we there yet? *Trends Neurosci. Educ.* **15**, 62–70 (2019).
26. A Harrewijn, et al., Combining fmri during resting state and an attention bias task in children. *Neuroimage* **205**, 116301 (2020).
27. F Li, et al., Brain network reconfiguration during motor imagery revealed by a large-scale network analysis of scalp eeg. *Brain Topogr.* **32**, 304–314 (2019).
